# Group interventions to improve health outcomes: a framework for their design and delivery

**DOI:** 10.1186/1471-2458-10-800

**Published:** 2010-12-31

**Authors:** Pat Hoddinott, Karen Allan, Alison Avenell, Jane Britten

**Affiliations:** 1 Health Services Research Unit, University of Aberdeen, Health Sciences Building, Foresterhill, Aberdeen AB25 2ZD, UK; 2 NHS Education for Scotland, Thistle House, 5th Floor, 91 Haymarket Terrace, Edinburgh, EH12 5HD, UK; 3 Centre for Rural Health, University of Aberdeen, The Centre for Health Science, Old Perth Road, Inverness, IV2 3JH, UK

## Abstract

**Background:**

Delivering an intervention to a group of patients to improve health outcomes is increasingly popular in public health and primary care, yet "group" is an umbrella term which encompasses a complex range of aims, theories, implementation processes and evaluation methods. We propose a framework for the design and process evaluation of health improvement interventions occurring in a group setting, which will assist practitioners, researchers and policy makers.

**Methods:**

We reviewed the wider literature on health improvement interventions delivered to patient groups and identified a gap in the literature for designing, evaluating and reporting these interventions. We drew on our experiences conducting systematic reviews, intervention, mixed method and ethnographic studies of groups for breastfeeding and weight management. A framework for health improvement group design and delivery evolved through an iterative process of primary research, reference to the literature and research team discussion.

**Results:**

Although there is an extensive literature on group processes in education, work, politics and psychological therapies, far less is known about groups where the aim is health improvement. Theories of behaviour change which are validated for individual use are often assumed to be generalisable to group settings, without being rigorously tested. Health improvement or behaviour change interventions delivered in a group setting are complex adaptive social processes with interactions between the group leader, participants, and the wider community and environment. Ecological models of health improvement, which embrace the complex relationship between behaviour, systems and the environment may be more relevant than an individual approach to behaviour change.

**Conclusion:**

The evidence for effectiveness and cost-effectiveness of group compared with one-to-one interventions for many areas of health improvement in public health and primary care is weak or unknown. Our proposed framework is the first step towards advocating a more systematic approach to designing, evaluating and reporting interventions in group settings, which is necessary to improve this currently weak evidence base. This framework will enable policy makers and practitioners to be better informed about what works, how it works and in which contexts when aiming to improve health in a group setting.

## Background

Groups are an alternative to individual encounters for health improvement, social support and changing behaviour, for example: smoking cessation [1]; weight loss [2]; parentcraft [3]; and self care for chronic conditions like diabetes [4] and osteoarthritis [5]. Such groups are evolving rapidly in response to cultural, epidemiological and environmental change, for example recent increases in cardiac rehabilitation groups [6], the expert patient programme led by trained patients with personal experience of a condition [7] and virtual internet self-help groups [8]. However the evidence for health improvement interventions delivered in group settings is dispersed through several systematic reviews of specific lifestyle behaviours, most of which focus on individual behaviour change interventions and theory. In our experience researching breastfeeding support and weight management groups since 2000 and 2002 respectively, we were surprised by the lack of guidelines for designing, evaluating or reporting health improvement interventions in group settings. We identified this as a gap to which our research could usefully contribute.

"Groups" feature in social, political, cultural, educational and work contexts, besides health, and have a variety of meanings, underlying theories and definitions. These are reviewed by Rupert Brown [9], a social psychologist, who proposes the following:

"A group exists when two or more people define themselves as members of it and when its existence is recognised by at least one other person or group of people who do not so define themselves"

The National Institute for Health and Clinical Excellence (NICE) behaviour change guidelines distinguish between interventions at the individual, community and population level [10]. Groups are included in the broad category of community interventions, defined as social or family groups linked by networks, geographical location or another common factor.

In this paper, we use a broad definition of health improvement to include health promotion, disease prevention (primary and secondary), public health, community development approaches and social support. We refer to "interventions in group settings" to define what happens to people within the group, the context in which it happens and the relationship between the two. Our definition of the setting refers to an observable health improvement group and the related activities and processes that occur. These include the place where the group meets and we consider the wider aspects of the space occupied by people and things, including the attached meanings and relationships [11]. It also includes the wider geographical, cultural, media, political and organisational environment of health improvement group settings.

Our aim is to provide a framework for the design and process evaluation of health improvement interventions in group settings, guided by the literature on designing complex interventions [12]. Detailed discussion of statistical aspects are not covered but are important as they need to take account of interactions between both group participants and group leaders [13]. Neither are specific methods of data collection for the process evaluation of group interventions discussed. Instead our framework poses a series of questions which are important to consider when designing and evaluating a group intervention. This is central to ecological theories of behaviour, as groups are complex systems with multiple interacting variables, at several levels which require a mixed method approach [14]. We recommend using a toolkit approach [15] to choosing the most appropriate methods (quantitative or qualitative) to answer each question, informed by existing evidence.

### Development of the framework

The framework has developed through our research into breastfeeding support and weight management groups, which included systematic reviews, mixed method group interventions and ethnographic studies [2,16]. Studies included a randomised controlled trial of a policy to provide breastfeeding groups across Scotland [17] with a mixed method evaluation of implementation processes [18]; a controlled intervention study of individual and group peer support for breastfeeding [19,20] and an ethnographic study of participant and provider experiences of weight management groups [21]. All of these studies conducted in-depth qualitative interviews with participants and providers and group observations.

In addition, our studies and this paper were informed by a literature review of health improvement interventions for patient groups. We searched Medline, CINAHL, Embase, The Cochrane Library, National Institute for Health and Clinical Excellence (NICE), Psycinfo and the International Bibliography of the Social Sciences using group$, class$, club$, workshop$ and program$ as title words for papers published between 1980 until 2009. This was a pragmatic search strategy to limit the number of papers identified and was necessary because of the widespread use of the word "group" in differing contexts in research papers. We identified systematic reviews in relevant areas, for example smoking and alcohol cessation; chronic diseases like diabetes, cancer support and heart disease and searched their reference lists. We also searched our personal reference archives and hand searched references in key papers. We excluded educational groups for teaching students or staff; organisational and management literature on work groups and teams, and treatment groups in mental health, where there is an extensive literature.

The framework evolved through an iterative process of mixed method data analysis, reference to the literature, reflection and research team discussion over a period of 6 years. It builds on reviews of group processes [9], small group work in education and work settings [22] and self-help or support groups [23].

### What is the evidence for health improvement interventions delivered in group settings?

The evidence for health improvement interventions in group settings is varied, reflecting their heterogeneity and complexity and some argue that policy for group interventions to encourage self care has raced ahead of the evidence [24]. Systematic reviews often focus on an individual disease, the type of treatment, or behaviour change theory and they often inappropriately combine the results of interventions delivered individually and in group settings in a meta-analysis [13]. For example, a Cochrane review of additional support for breastfeeding differentiates between whether the support is lay or professional, but not between interventions delivered to individuals or groups [25]. In a systematic review of smoking cessation groups, theories of behaviour change validated for individual delivery, for example stages of change or cognitive behavioural theory, have been assumed to be transferable to delivery in a group setting [1]. This systematic review of smoking cessation interventions [1] is one of few which has specifically analysed outcomes for interventions in group settings and compares them with self-help materials, individual counselling, nicotine replacement or no intervention. It concludes that group programmes are more effective than no intervention or self help interventions, but there is insufficient evidence to evaluate whether groups are more effective than individual counselling. Another systematic review comparing group versus individually delivered interventions for weight management in adult obesity found that group interventions report significantly more weight loss at 1 year follow-up compared with the same intervention individually delivered [2]. A systematic review of group education programmes for adults with type 2 diabetes compared with routine treatment, waiting list control or no intervention, found group programmes were effective at lowering glycated haemoglobin, fasting blood glucose, blood pressure, weight and medication use [4]. For cardiac rehabilitation, interventions delivered individually at home were as effective as those delivered in a centre to a group [6].

The aims of a group intervention, the underlying behavioural change theories and group processes can determine who attends and both the group and the individual health outcomes. A one size fits all approach seldom meets everyone's needs and it has been argued that for self care in chronic disease, groups should only be considered for simple standardised messages, where peer support is beneficial or preferred and where a group will save money [24]. Importantly, some groups may increase health inequalities by attracting more educated and higher income participants [17,26,27]. It has been suggested that support groups are more likely to be sought for diseases viewed as stigmatizing, like AIDS, alcoholism, breast and prostate cancer rather than less stigmatizing, yet important, diseases like heart disease [28]. There are inconsistencies in self care group definitions, for example in a survey of American self care groups 60% of those identified had professional facilitators and the clinical potential for social support in combination with professional guidance compared to peer only support is largely unknown [28].

The potential for cost savings with group compared to individual health improvement interventions can appear attractive; however the health economic evidence is mixed and often weak. In a review of individual or group self care interventions, there was insufficient evidence to support the claim that group interventions are cheaper and there are often trade-offs between the numbers of patients treated and the quantity of intervention each individual receives [24]. Smoking cessation groups are no more cost effective than intensive individual counseling [1] and breastfeeding groups provided as part of routine care would have similar costs to individual health visitor home visits to group participants [17]. Group interventions for weight management in obesity are potentially more resource saving in terms of total health professional-hours involved per participant [2]. Few self care group interventions for arthritis have measured service use and findings are conflicting [29,30].

### Why do we need a framework?

In the literature discussed above, it is clear that the jury is still out when it comes to deciding whether group or individual interventions are more effective and cost-effective at improving health outcomes. Group processes and interactions have received less attention than dyadic or individual behaviour change mechanisms and little is known about which components of groups contribute to effectiveness. As a result there are many unanswered questions for practitioners and policy makers who aim to establish patient groups to improve health outcomes. The NICE behaviour change guidelines recommend being as specific as possible about the content of the intervention, spelling out what is done, to whom and in what social and economic context [10]. A challenge with groups is to unpick the extent to which outcomes are determined by leadership style, personality, participants' characteristics or more complex interactions. Having a framework for design and process evaluation is one step towards producing this evidence and we describe this below.

### The place, setting and context of group interventions

The first and crucial stage in designing and evaluating a group intervention is to consider how aspects of the setting will impact on all aspects of group processes, composition and outcomes (Table 1). In a trial which randomised primary care organisations to deliver a policy to provide breastfeeding support groups, the breastfeeding outcomes were explained by the characteristics of the primary care organisation, including health inequalities and deprivation, the amount of organisational change taking place and multi-disciplinary teamwork [18]. The breastfeeding outcomes could not be explained by the amount of intervention delivered or the number of people attending the groups.

**Table 1:** The setting

**A) Within the meeting place and venue - the activity setting** **B) The immediate surrounding environment including parallel activity settings** **C) The wider geographical area** ***For each of the above consider:*** ○ Socio-demographic characteristics, facilities, human and structural resources (e.g. funds, time, people, physical objects) ○ Access issues ○ Sensory aspects (comfort, temperature, noise, smell, visual) ○ Meanings attributed to the setting including opportunities and threats **D) The wider policy, political, media, legal, cultural and environmental context and how it interacts with the group**

Evaluating how different environmental settings influence behaviour has been relatively neglected in research compared to the predominant approach of measuring the psychological variables of individuals receiving a behaviour change intervention. Theorising interventions as events in complex systems is particularly relevant to health improvement groups, where the context, the change in relationships and resources over time are important [31]. The importance of the behavioural setting [32] or the activity setting [33] as a unit of analysis is a cross disciplinary feature of psychological, social and biological ecology [32,34]. Behavioural or activity settings refer to time and space bounded patterns of behaviour, and the concept originates from research which characterised all behaviour settings in a small American community [32]. This demonstrated that behavioural settings have effects on individuals that extend beyond the specific behaviour demanded by the activity.

It can be helpful to consider the context in which the group intervention takes place at macro, meso and micro levels, as proposed in ecological models of health improvement [35]. The macro level considers how wider policy, economic and socio-cultural factors interact with the group, for example media scares or the cultural values of participants. The meso level includes the interrelations between the group, the setting and the surrounding environment, for example holding the group in an affluent or disadvantaged area can influence perceptions and who attends a group [18]. Importantly, the impact of parallel activity settings [33] which may target the same population should be considered. For example, the impact that general parent craft groups open to all women can have on participation in breastfeeding only groups [18]. Similarly, consideration should be given to assessing the impact of activities displaced by the group intervention [31]. What providers and participants stop doing when a health improvement group starts is likely to be crucial to outcomes, yet this is seldom systematically described or evaluated. The micro level is the interaction between group participants and the space where the group meets. Community settings like church halls or family centres convey different meanings for people compared to health settings and health service group leaders may behave differently outside health service settings [18]. Group resources can vary between purposely designed venues with state of the art props to "make do" multi-purpose clinically cluttered spaces. Consideration should also be given to sensory perceptions including comfort, temperature, noise, smell and visual appearance [20].

### Designing a group intervention

Table 2 focuses on the design of the intervention, the theory underlying the choice of intervention, the target population and choosing the relevant behavioural outcome to measure.

**Table 2:** Designing interventions in group settings

**What is the intervention and what quantity will be delivered?**
○ The group itself as the intervention
○ The group leader delivers the intervention
○ The group as a vehicle for delivering the intervention to a wider population
○ The group size, frequency, duration and lifetime
**How does someone become a group member?**
○ Are there gatekeepers and how do they operate
○ Self or professional referral, with or without criteria
○ Advertising: general or targeted
○ Access *to attend *meetings: open (anyone can drop in and out of attending meetings); closed (membership registration on attendance, in advance or for a fixed period)
○ Access *during *a group meeting: open (drop in and out); closed (fixed start and finish)
○ Barriers, facilitators and entry rituals
○ Incentives and costs (financial and non financial) - joining, recurring, optional, refundable
**What social and behaviour theories inform the intervention?**
○ Education: factual, tacit or experiential knowledge
○ Support: for a specific behaviour, attitude or belief
○ Cognitive approaches: to change thinking about a behaviour
○ Performing a behaviour or activity
○ Rewarding a behaviour or group attendance
○ Competition between groups or group members
**How are the group influencing attitudes, beliefs and behaviours? For example:**
○ Social comparison theory
○ Social support theory
○ Social learning theory
○ Social impact theory
**What are the outcomes?**
○ Initiate or sustain a desired behaviour
○ Reduce, stop or prevent a relapse of an undesirable behaviour
○ Substitute a desirable for an undesirable behaviour
○ Change how an existing behaviour is enacted
○ Change attitudes or beliefs which might predict or mediate a behaviour e.g. self-efficacy
**What is the target population for intervention delivery and outcome measurement?**
○ Who is targeted? People with specific behaviours, socio-demographic characteristics or diseases; from particular geographic areas or organisations
○ Whose outcomes will be measured? Individual group attenders, pooled group outcomes, wider population

#### What is the intervention and what quantity will be delivered?

The interactions between group members may form the intervention as with peer support or self-care [7]. The intervention might be something which is delivered by the group leader, like a particular diet or exercise programme and there may be a range of intended and actual interaction between group participants from minimal, mainly non-verbal communication to high level engagement. The intervention may intend to have an outreach beyond the group setting, for example sex education delivered by peer educators [36]. It is particularly important in trials of interventions in group settings to consider the statistical aspects of whether the group attributes and processes detailed in Tables 1, 2 and 3 are acting as mediators or moderators between the intervention and the health outcomes. The group size, frequency, duration and lifetime will all influence group composition and processes. Decisions about the components of the intervention are linked to the underlying theory informing it and the outcomes of interest which are discussed below. There are detailed guidelines about how to report the attributes of complex interventions which are relevant to groups [37,38]. However, reducing complex social processes like groups into standardised, reproducible intervention components has its critics [39] and interventions which are responsive to local contexts and change over time may be more appropriate.

#### How does someone become a group member?

It is important to assess how entry rituals and gatekeeper assumptions are influencing information dissemination and recruitment to a group. Some groups have elaborate entry rituals, for example general practitioners may be asked to complete forms prior to registering for a cardiac rehabilitation group whereas other groups encourage open access. Convenience, minimising barriers and ensuring that the participant benefits outweigh the risks are crucial to a successful group and will be highly context dependent [20]. In a study comparing commercial and health service groups for weight loss, some participants valued the flexibility and autonomy offered by groups where you could drop in just to be weighed and leave with minimal group interaction whereas others preferred leader facilitated discussion [21]. Relationships between professional gatekeepers and "the group" can both facilitate or hinder attendance, for example infrequent midwife participation led to limited attendance by pregnant women in a trial of breastfeeding support groups [18].

#### How will the group influence people?

This is a contentious area as no single theory can capture the complexity of intra and inter group behaviour. In Table 2, we suggest separating the social and behavioural theories that inform the group intervention from the theory of how the group itself is likely to influence the attitudes, beliefs and behaviours of participants. Individual behaviour change theories and techniques have been reviewed [40], but not specifically for delivery in group settings and it cannot be assumed that they are generalisable, as individuals can behave differently when in a group [9]. Group composition may have a causal effect on group outcomes; the group may be the social context which allows other inter-personal psychological phenomena to unfold or group composition may be a consequence of other external factors [41].

How intra and inter group processes influence people's attitudes, beliefs and behaviours are reviewed elsewhere [9], although not specifically in the context of health improvement. In Table 2 we provide some examples of theories which are particularly relevant to health improvement group interactions. Festinger's Social Comparison Theory [42] proposes that conformity within a group is dependent on three main motivations: dependence on others for information to self-evaluate; achieving group goals and the need for approval and a desire not to seem different. Festinger hypothesises that group participants will try to improve their performance and will differ in whether they compare upwards or downwards, for similarities or dissimilarities. They will select different attributes to compare, which may not always be the expected ones [20]. Downward comparison where a person wants to know how dissimilar he/she is from the most undesirable person is more common in the expert patient programme and serves to protect a threatened self-esteem [27]. Festinger hypothesises that high status members are motivated to try and improve the performance of others less capable or they might perform below their capability so that they would not appear too different from the rest. Criticisms are that social comparison theory focuses on intra-group comparisons, whereas temporal comparisons with the past or the future, or comparisons with other groups or non group participants may be of equal importance [9].

Social support theory proposes that information is disseminated more effectively between networks of people with strong social ties and this confers health benefits [43]. In groups, strong and weak social connections may differ in their effects and either reinforce existing attitudes and behaviours or mediate change. Influence often extends beyond the group to the family, local community or population and this may be intentional, for example in peer education interventions, or unintentional. This poses challenges for outcome measurement and contamination, as the cumulative outcome is the sum of the individual outcomes and the collateral positive or negative health outcomes of others [44]. There is a continuing debate about the relative importance of social support network size and the strength of connections. On-line support groups are becoming increasingly popular and provide large networks of relatively superficial support [28]. Further comparisons between actual and virtual health improvement and support groups are warranted.

Bandura's Social Learning Theory with its emphasis on learning through observation and modelling behaviour [45] is particularly relevant to behaviours involving action or performing, like breastfeeding or parenting skills [20]. Minorities and majorities both influence group processes and Social Impact Theory proposes a negatively accelerating continuum of influence based on observations that the first social stimulus has the greatest effect, the second less effect and the third less still [46].

#### What are the health outcomes and target populations?

Outcomes from interventions delivered in a group are usually measured at the individual level [1-3], and most studies do not consider interactions between patients in the same group which may lead to correlation of outcomes [13]. Cluster randomized controlled trials where randomization and outcome measurement have occurred at the group level are rare. Occasionally wider population level outcomes are measured, for example the multifaceted STD/HIV Intervention Project (SHIP) where peer educators delivered individual and group interventions resulting in dramatic and sustained improvements in sexually transmitted infections at a population level [36]. As with cluster randomized trials, individuals in a group cannot be considered to be independent of each other and variation in the outcome is likely to be smaller for participants treated in the same group than for participants treated in different groups. Similarly, if the same group leader delivers the intervention to different groups of participants, the outcome may differ less than the outcome for participants treated by different group leaders. This design clustering is an additional consideration when deciding whether to randomise individuals or clusters to a group intervention.

### What happens within a group?

Table 3 proposes a series of questions examining the micro-level of interactions between the group leader, new and existing group members and the group setting. It is closely related to the ecological, social and behavioural theories described above. Group processes and their interactions with the group setting can determine group survival and the intended outcomes, but they have been under-researched in the context of health improvement groups and the relative importance of individual components is unknown. Group Environment Scales (GES) have been developed to systematically measure the norms, values and psychosocial characteristics of social environments [47]. The underlying theory is that environments like people have unique personalities, climates or atmospheres which are important determinants of behaviour. GES have three basic dimensions: *relationship dimensions *which assess the extent to which individuals are involved, for example cohesion, support; *personal development dimensions*, for example autonomy, goal attainment; *system maintenance or system change dimensions *for example order, organisation, clarity, control. GES have been developed in the field of mental health treatment, education and work and little is known about their generalisablity to health improvement groups. They have been used to compare self-help groups [23] and these three dimensions seem salient in a wide variety of social settings, although the characteristics of each dimension may vary [34,48]. The dimensions we use in Table 3 are adapted from GES to provide a better fit with our data. In particular the *personal development dimension *seems more relevant to educational or treatment groups and we have reframed this as *attributes of the leader and the group participants*. As groups are complex systems there is some overlap and interaction between dimensions.

**Table 3:** What happens within a group

**System maintenance:**
○ Who organises and leads the group? Is he/she internal or external to the group? How is he/she appointed or elected?
○ The leader's role in initiating, planning, setting up, organising and running the group
○ One or several group leaders/co-leaders? Similar or complementary leader attributes; continuity or rotation of leadership; fixed or flexible?
○ Is group content: flexible; repetitive; different over time; leader or participant determined?
○ Is there group member feedback? Formal or informal? How does feedback change group processes?
**Leader attributes:**
○ Socio-demographic characteristics, professional qualifications, training, personal experience of the behaviour or problem, interpersonal communication skills
○ To what extent is the leader able to attend to both the group task and the socio-emotional aspects of the group?
○ What is the leadership style: directive/nondirective; proactive/reactive; led (hierarchical)/facilitated (co-operative)/present (autonomous)?
○ How flexible is the leader and how does the leader change over time?
○ What are the benefits/rewards and costs/burdens of being a leader and how are they manifest?
**Relationship between the leader and group:**
○ How does the leader have legitimacy in the eyes of the group: e.g., expert knowledge; skills; competence; personal attributes; personal experience; conforming to group norms; acting fairly; group identity; geographical residence?
○ What techniques does the leader use: education; persuasion; providing a practical task or service; advocacy; advising: supporting; empowering; counselling; listening; providing vision; inspiration or motivation; selling?
○ What do the group initiators, leaders and group members view as the purpose (aims and objectives) of the group? How similar or different are their perspectives?
**Attributes of the group participants**
○ To what extent are the group task/goal or socio-emotionally orientated?
○ To what extent are there shared goals?
○ What does it mean to be a group member/non-member in terms of personal and social identity?
○ Do participants categorise themselves; adopt specific group roles or a hierarchical status?
○ What is the level of anonymity or public performance within the group? High with each individual speaking in turn or low as in a crowd where anonymity can be maintained?
**Group relationships**
○ To what extent are socio-emotional interactions positive or negative?
○ How do intra-, inter-group and non-group member relationships change over time?
○ Do group attitudes, beliefs and behaviours become more or less extreme over time?
○ How similar or different are the attitudes and behaviours of group members?
○ What are the group norms, how are the limits of acceptable behaviour defined and is difference tolerated?
○ Do the group norms encourage or inhibit goal attainment and/or positive socio-emotional interactions?
○ How cohesive is the group?
○ How much communication between group members occurs? Minimal, mostly non-verbal to in depth engagement?
○ Is communication within the group channelled through the leader, within subgroups or free with multiple conversations?

We have used the term "group leader" to embrace a range of observed styles which others have defined [9,22]. Variation in the delivery skills of group leaders can theoretically determine the success of the intervention and it is important to consider the statistical effects of such clustering in the design [13]. Education, support and behavioural interventions in a group setting are characterised by different communication styles. For example, a group leader may be an advocate of a method, philosophy, activity, seller/deliverer of a "product" or a facilitator of person centred approaches, for example empowerment, counselling or support [49]. How leaders with personal experience of a condition, for example in commercial weight management groups [21] and the expert patient programme [7], influence group outcomes is largely unknown. Some groups are multifaceted with communication interspersed with activities like relaxation, physical activity, weighing or physiological monitoring. Health service training in group leadership is variable depending on the type of group and the nature of the intervention. For example, exercise groups have standardised qualifications for leaders, whereas in some primary care groups for weight loss, practice nurses are self-taught and learn on the job [21].

The NICE behaviour change guidelines [10] recommend that community interventions should: improve self-efficacy; develop and maintain supportive social networks; promote resilience; build skills; promote participation in voluntary activities; promote involvement in planning and delivery of services and have access to the financial and material resources needed to facilitate behaviour change. Of fundamental importance is having a clearly stated purpose with aims and objectives that are agreed by everyone. However, even with clear aims and objectives, differing interpretations will arise. For example, in a trial of breastfeeding support groups, leaders interpreted the word "support" in different ways. Despite a clear protocol stating that support should be woman-centred and based on informed choice, some interpreted "support" as promoting and encouraging exclusive breastfeeding as you might support a football team [16]. Some experts classify groups into either task orientated groups, where individuals or the group have specified goals, or socio-emotional groups where feelings and inter-personal relationships are paramount [9]. However, our group observations suggest that this binary classification may not apply to the complexity of health improvement interventions, which usually combine task and socio-emotional objectives [16,21].

A study of cancer support groups illustrates how an analysis of group processes can provide important evidence and highlights the difficulties with pre-selecting group composition [50]. Education and discussion groups combining distressed and undistressed cancer patients were compared with a group of distressed patients. The heterogeneous group increased the social comparison opportunities for distressed patients and benefited them. However, undistressed women with breast cancer who had high levels of social support showed a slight deterioration in physical functioning when attending the heterogeneous group, which raised ethical concerns. The authors suggest training undistressed participants to maximise their benefit for distressed participants but to minimise negative consequences for themselves.

Individuals tend to conform to the attitudes and behaviours of the majority within a group, and cliques can develop. For example, some breastfeeding groups can be dominated by mothers breastfeeding older infants, which may be off-putting to new mothers joining, but an over-all "feel-good atmosphere" can override personal differences [20]. Studies suggest that conformity and uniformity in groups increases with group size and over time and groups tend to exhibit more extreme attitudes, beliefs and behaviours than the individual group participants [9].

## Conclusion

The evidence for effectiveness and cost-effectiveness of group compared with one-to-one interventions for health improvement is weak or unknown. Health improvement or behaviour change interventions delivered in a group setting are complex adaptive social processes with interactions between the group leader, participants, and the wider community and environment. As with any complex intervention, there is a need to understand which aspects of group processes work, for whom, how and in what circumstances. Ecological models of behavioural change may be more appropriate than applying theories of individual behavioural change in a group setting. This framework is the first step towards advocating a more systematic approach to designing, evaluating and reporting interventions in group settings, which is necessary to improve the currently weak evidence base.

## Competing interests

The authors declare that they have no competing interests.

## Authors' contributions

PH had the idea for the paper and drafted it. All authors contributed to the analysis of data in the ethnographic studies of interventions in group settings which informed this paper and to writing the paper. All authors read and approved the final manuscript. PH is guarantor.

## Pre-publication history

The pre-publication history for this paper can be accessed here:

http://www.biomedcentral.com/1471-2458/10/800/prepub
